# The impact of their role on telephone crisis support workers' psychological wellbeing and functioning: Quantitative findings from a mixed methods investigation

**DOI:** 10.1371/journal.pone.0207645

**Published:** 2018-12-19

**Authors:** Taneile Ashlea Kitchingman, Peter Caputi, Alan Woodward, Coralie Joy Wilson, Ian Wilson

**Affiliations:** 1 School of Psychology, University of Wollongong, Wollongong, New South Wales, Australia; 2 Illawarra Health and Medical Research Institute, University of Wollongong, Wollongong, New South Wales, Australia; 3 Lifeline Australia, Canberra, Australian Capital Territory, Australia; 4 Centre for Mental Health, Melbourne School of Population and Global Health, University of Melbourne, Melbourne, Victoria, Australia; 5 School of Medicine, University of Wollongong, Wollongong, New South Wales, Australia; University of Birmingham, UNITED KINGDOM

## Abstract

Research suggests that frequent empathic engagement with others in distress places helpers in registered professional roles (e.g. medical practitioners, psychologists) at risk of functional impairment related to symptoms of psychological distress, including the delivery of sub-optimal care to patients. Preliminary research suggests that telephone crisis support workers may also be impacted in a similar way. This repeated measures study is the first known research to examine telephone crisis support workers’ functional impairment related to symptoms of psychological distress before and after speaking with callers in crisis. A representative sample of telephone crisis support workers from Lifeline Australia participated by completing three surveys: 1) directly before; 2) directly after; and 3) one week after completing a shift on the national crisis line. Surveys included standardised measures of functional impairment, psychological distress, lived experience of mental health issues and suicide, motivations for volunteering, coping strategies and help-seeking. Categorical items were used to assess personal and shift-related factors. Repeated measures analyses of variance were used to identify changes in symptoms of psychological distress and impairment across time points. Structural equation modelling was used to test relationships within a hypothesised model of impairment. A significant proportion of participants reported functional impairment related to symptoms of psychological distress. Significant differences in functional impairment and symptoms of psychological distress were detected, and were associated with different mechanisms, across time points. An important outcome of this study is empirically-supported models which explain how telephone crisis support workers come to experience functional impairment in relation to their TCS role, as well as other work/study, home/family and social/leisure activities. Results warrant the deliberate development and/or modification of existing service strategies to optimise telephone crisis support workers’ psychological wellbeing and functioning, including by structuring the work environment and emphasising certain messages during training and supervision.

## Introduction

Crisis intervention strategies that include readily accessible support without referral requirement are fundamental mental health strategies. Telephone crisis support services have the capacity to provide immediate support for rapid intervention. Within Australia, non-clinical telephone crisis support services provide essential infrastructure for individuals in crisis. Crisis lines meet gaps in other services to support the community, and are a viable alternative for people who would otherwise not receive support during their crises. These services are effective in attracting people seeking help, especially those experiencing severe mental health issues and/or thinking about suicide [1–3]. Research shows that contact with telephone crisis support services significantly improves the help-seeker’s mental state, reduces their suicidality, and often facilitates engagement with interventions that can offer longer term benefits [4–6]. Individuals in crisis continue to access telephone crisis support services at an increasing rate. For example, Lifeline Australia Telephone Crisis Supporters (TCSs) answered 821,804 calls in the 2015 financial year, representing an increase of 12% from the 2014 financial year, and 33% from the 2013 financial year [7].

Optimal crisis intervention requires workers’ delivery of support to those in crisis by implementing a proven service model. While Australian telephone crisis support workers are trained in evidence-based, best practice crisis intervention models, much less is known about how this section of the mental health workforce are performing, including whether they are resilient to occupational hazards. Of particular consideration is the factor that telephone crisis support workers are frequently exposed to others who are highly distressed and suicidal [4, 5, 8].

A key feature of the crisis support service model is empathic engagement with callers to foster a connection that will provide emotional support and facilitate the help seeking experience. However, empathic engagement with others who are highly distressed and suicidal can lead helpers to experience elevated symptoms of psychological distress [9–12], help-negation [13–16], functional impairment [17, 18] and to deliver sub-optimal care to patients [19–21]. To date, research has focused on helpers in registered professional roles (e.g., mental health and medical professionals), suggesting that the impact of frequent empathic engagement with distressed others on workers performing non-professional roles, including telephone crisis support workers, may have been overlooked.

Due to the nature of their role, telephone crisis support workers also experience unique stressors. For example, they are unable to observe non-verbal communication cues or anticipate or control the types of contacts they receive, and are also unable to track changes in the help-seeker due to the ‘one-shot’ nature of contact with these services [22]. In addition, technology-based crisis workers generally have less training to prepare them for the role and are less likely to have access to supervision and ongoing professional development than workers in registered professional roles [23]. Most telephone crisis support workers are also volunteers who are not paid for their time [24].

In acknowledgment of their exposure “to high risk, stressful and challenging situations”, a number of suicide prevention organisations in Australia that have met as the National Coalition for Suicide Prevention have reported that crisis responders “need to be trained and supported to manage their own needs, as well as the needs of those they respond to” [25]. However, conclusions about how telephone crisis support workers may be impacted by frequently speaking with highly distressed and suicidal callers are unable to be drawn due to the paucity and methodological limitations of current data [26]. Preliminary research suggests that a significant proportion of telephone crisis support workers experience functional impairment related to elevated personal symptoms of psychological distress [27]. Cross-sectional research supports a model of telephone crisis support workers’ functional impairment whereby emotion regulation difficulties, personal symptoms of psychological distress and low intentions to seek help for personal symptoms interact to influence workers’ ability to manage their day-to-day activities [27].

One limitation of these preliminary studies is that they have failed to acknowledge research indicating that telephone crisis support workers’ psychological state changes from before to after completing a shift on the crisis line [28]. Another limitation of previous studies is that they have employed a global measure of functional impairment. Most telephone crisis support workers are volunteers who are also engaged in other study or work, have family responsibilities and social lives. The current study builds on previous research by identifying mechanisms of telephone crisis support workers’ impairment in each of these functional domains during the week before and the week after completing a shift on the crisis line. The findings of this study may inform the development and/or modification of existing service recruitment, training, assessment, self-care, supervision and support strategies to optimise telephone crisis support workers’ psychological wellbeing and delivery of support to callers.

### An extended model of telephone crisis support workers’ functional impairment

#### Negative affect

Empathy is an essential factor in effective helping [29, 30] and accounts for approximately 10% of the variance in psychotherapy outcomes [31]. In the last decade neuroscience has also identified empathy as a primary conduit for helpers’ development of personal distress [32–34]. Neuroimaging studies demonstrate that when we engage with others in distress, ‘affect sharing’–a bottom-up process that has been attributed to the human mirror neuron system–takes place [32, 35, 36]. Observing another person in a particular affective state (e.g., fatigued, confused, tense, dejected, angry) activates the helper’s mirror neuron system, causing the helper to experience similar affect, and related autonomic and somatic responses [37]. Listening to and envisioning another in distress also activates the mirror neuron system [37]. By engaging empathetically with crisis callers in the absence of non-verbal information, it is likely that telephone crisis support workers experience heightened mirror neuron activation, and stronger subsequent affective, autonomic and somatic responses, increasing their risk for personal distress. On the basis of this review, it is hypothesised that negative affect will predict psychological distress (H1).

#### Personal and shift factors

Research has identified a number of personal and shift factors which increase helpers’ risk for distress. Personal risk factors include younger age [16, 38, 39], female sex [9, 16, 39, 40, 41], regional/rural location [42, 43], lower levels of educational attainment [11] and experience in helping roles [11, 28, 44, 45], lived experience of mental health issues and suicide [38], and self- rather than other-oriented motivations for helping [46]. Together with their frequent engagement with highly distressed callers [4, 5, 8], these factors may increase telephone crisis support workers’ vulnerability to personal distress.

A study that examined levels of perceived stress among telephone crisis support workers on a suicide prevention line found that levels of perceived stress during the shift was related to having spent a greater amount of time speaking to callers during the shift [29]. Level of stress after completing the shift was also related to total length of time spent on calls, as well as the number of other people present at the suicide prevention centre during the shift [29]. Based on this evidence, it is hypothesised that personal (H2) and shift factors (H3) will predict psychological distress.

#### Psychological distress

While elevated symptoms of distress do not necessarily result in functional impairment [47], they often precede functional impairment and are considered a “warning signal” [48]. Researchers and clinicians converge on the conclusion that symptoms of psychological distress may adversely affect functioning globally, as well as in specific domains such as work, home and social functioning. The DSM makes functional impairment an explicit part of the criteria for establishing a diagnosis [49], and clinical practice guidelines state that a primary goal of mental health treatment is restoration of functioning [50]. Research which has assessed domain-specific functional consequences of symptoms of psychological distress suggests that symptoms and functioning hold a tenuous relationship requiring both to be measured routinely. While most researchers and clinicians recognise these implications, much focus has been given to the assessment and resolution of symptoms of psychological distress, with relatively little attention given to functional improvements [51, 52]. Therefore, researchers are encouraged to re-prioritise their measure selection to shift the focus away from mere symptom assessment toward a comprehensive measurement model that includes functional outcomes [52].

Research conducted with professional helpers has documented the impact of symptoms of psychological distress on personal and professional functioning, including the inability to communicate or use key skills effectively [20, 21], and the delivery of sub-optimal care [16, 19, 53, 54]. More than half (60%) of all psychologists in a seminal study reported having practiced therapy when they were too distressed to be effective [53]. Physicians’ personal symptoms of distress have also been associated with inadequate care practices, ranging from minor mistakes to potentially serious medical errors [21, 55]. Preliminary results suggest that telephone crisis support workers also experience functional impairment related to symptoms of psychological distress. A significant proportion of telephone crisis support workers who participated in one study reported moderate to very high symptoms of psychological distress, and these participants reported significantly greater functional impairment than those who reported low level symptoms [27]. In keeping with this research, it is hypothesised that psychological distress will predict impairment (H4).

#### Coping and help-seeking

Coping and help-seeking are established as key variables in the process of reducing, minimising or tolerating stress, and in preventing psychological distress from progressing to functional impairment [56]. The American Psychological Association-endorsed stress-distress-impairment continuum [57] describes the likely progression from distress to impairment for helpers who do not pursue or receive appropriate ameliorative effects to interrupt this progression. It posits that those helpers who use inappropriate or ineffective means to manage their distress are at increased risk of impairment, including compromised functionality, sub-optimal service delivery, and inappropriate or unethical behaviour.

Research suggests that, while helpers believe in the usefulness of seeking help, and engaging activities to prevent or reduce distress and impairment, this belief is not associated with time spent engaging in such strategies [58]. Helping professionals report that they are unlikely to seek help for elevated personal symptoms of distress, especially from another professional [13–16]. They also report the use of maladaptive coping strategies such as magical thinking, detachment and feeling personally responsible, which increase their risk of distress [28]. On the basis of this research, it is hypothesised that help-seeking (H5) and coping (H6) will moderate the relationship between psychological distress and impairment.

**Fig 1:**
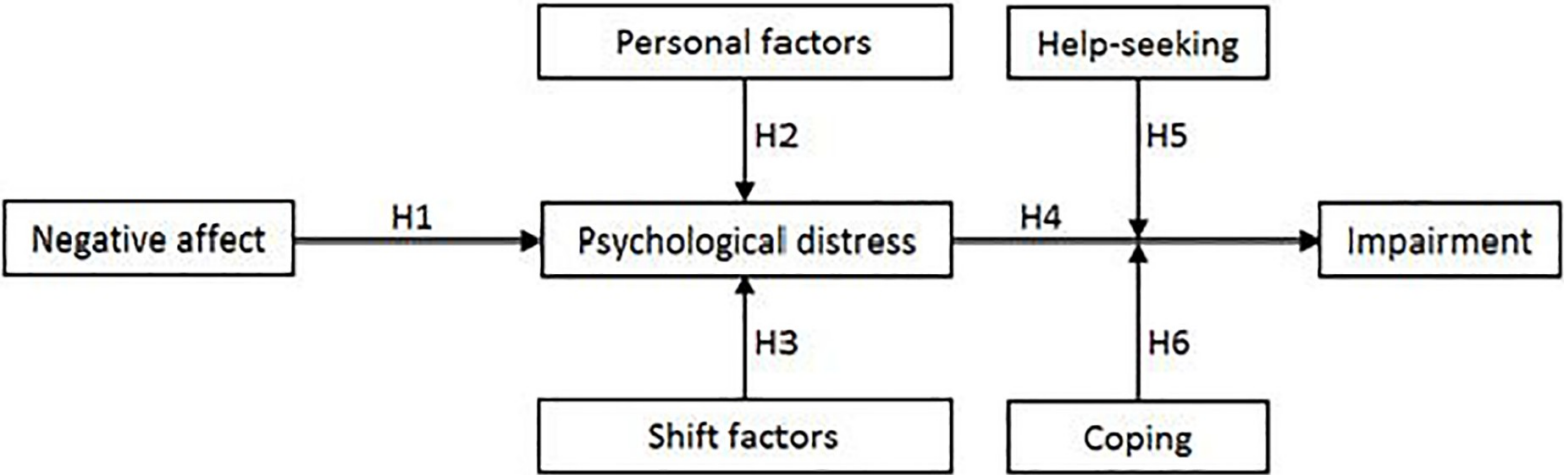
Hypothesised model of functional impairment among telephone crisis support workers.

Hypothesised relationships are presented in Fig 1.

## Materials and methods

### Design

A concurrent, mixed methods design [59] was used to examine telephone crisis support workers’ functional impairment related to symptoms of psychological distress. The purpose of this design was complementarity; that is, using quantitative and qualitative techniques to measure overlapping but also different aspects of the phenomenon of interest, functional impairment. Only the quantitative component of the study is reported in this paper.

The quantitative component of the study was designed in accordance with the STROBE criteria for cross-sectional studies [60, 61]. The completed STROBE checklist has been submitted as S1 Table.

### Procedure

Participants completed three surveys. They completed Survey 1 online directly before commencing their shift on the crisis line. In keeping with existing service procedures, they completed a call summary sheet for each call taken during the shift (See Measures–Shift factors). Participants completed Survey 2 online directly after completing their shift on the crisis line. Exactly one week following their shift, participants received an email containing the electronic link to Survey 3, which they completed online from a place of their convenience. A subset of participants also volunteered to participate in an interview, the results of which are reported elsewhere.

### Participants

Participants were recruited from the largest volume telephone crisis support service provider in Australia, Lifeline. In preparation for the main study, a table was created listing the location and number of TCSs working at the 42 crisis support centres in Australia. Centre locations were categorised as metropolitan or regional according to the Australian Standard Geographical Classification system [62]. Three centres from different location categories were selected to participate in the study in order to ensure representativeness.

Participants were recruited directly by the first author, who visited each of the three selected centres over separate two-week periods during May and June 2016. Each TCS who completed a shift on the crisis line during the recruitment period was invited to participate in the study. Participants provided informed consent (written), understood that their participation was voluntary, and their responses would be identifiable only by a unique code number.

They were provided with the contact details for various mental health resources, including physician directory information, helplines, informational resources and online services, which they were encouraged to access if they were concerned about their wellbeing. The study was approved by the University of Wollongong Human Research Ethics Committee.

Power calculations were used to determine that a minimum of 50 participants was required to detect an effect size of .25 with .80 power and .05 error. In total, 124 of the 154 TCSs who completed shifts during the recruitment period participated in the study by completing at least one survey, representing a response rate of approximately 81%. Data provided by 14 participants was excluded, as these participants reported completing more than one shift on the crisis line per week, leaving a final sample of 110 TCSs. Eight (7.3%) of these participants did not complete Survey 2, and 19 (17.3%) did not complete Survey 3.

Participants ranged in age from 19 to 84 years (*M* = 48.75, *SD* = 15.01). On average, participants had been in the TCS role for approximately 4 years (*M* = 3.74, *SD* = 3.78, Range = 1–17 years). Further demographic characteristics are provided in Table 1. Confirmation of the representativeness of the study sample in terms of age, gender and role type was established by comparison with recent service operational data [63, 64] which indicate that 76% of workers are female, 50% are aged 35–50 years, and 7.7% have a paid role.

**Table 1:** Demographic characteristics of study participants.

		Total *n*(%)
Gender	Male	26(23.6)
	Female	80(72.7)
Relationship status	Single	21(19.1)
	In a relationship	14(12.7)
	De facto	7(6.4)
	Married	51(46.4)
	Separated	3(2.7)
	Divorced	9(8.2)
	Widowed	1(0.9)
Highest educational qualification	Year 10/equivalent	5(4.5)
	Year 12/equivalent	5(4.5)
	Apprenticeship/equivalent	5(4.5)
	Diploma/equivalent	31(28.2)
	Undergraduate university degree	39(35.5)
	Postgraduate university degree	21(19.1)
Country of birth	Australia	82(74.5)
	Other English speaking country	19(17.3)
	Other non-English speaking country	5(4.5)
Centre	1 –Metropolitan	20(27.3)
	2 –Regional	39(35.5)
	3 –Regional	37(33.6)
Role	PTCS only	47(42.7)
	PTCS plus volunteer role	2(1.8)
	PTCS plus paid overnight shifts	2(1.8)
	TCS only	40(36.4)
	TCS plus volunteer role	11(10.0)
	TCS plus other paid role	3(2.7)

*Note*. PTCS = Probationary Telephone Crisis Supporter. TCS = Telephone Crisis Supporter.

Percentages for each characteristic do not add up to 100% where participants chose not to respond to the corresponding survey item.

Prior to the main study, a pilot study was conducted with 28 TCSs to pre-test the feasibility of the study design and reliability and validity of study measures. Evidence suggests that a sample size of 25 is required for pilot studies with aims related to instrumentation [65]. Participants in the pilot study were excluded from the main study.

### Measures

#### Functional impairment

The Sheehan Disability Scale [66] includes three items which measure functional impairment in the inter-related domains of work/school, social life/leisure activities, and family life/home responsibilities. Participants in the current study were asked to rate the work/school item in relation to their work/study outside of Lifeline, if applicable. A fourth item was added to represent the domain of work as a TCS. Participants rated the extent to which their symptoms impaired their regular activities in each domain over the past week on a Likert scale ranging from 0 *Not at all* to 10 *Extremely*. Individual subscale scores greater than 5 suggest significant impairment. Subscale scores can also be combined into a single score indicating global functional impairment, with a score of 15 indicating significant impairment. Two additional SDS items asked respondents how many days within the past week their symptoms caused them to: 1) miss; and 2) be less productive in activities within each domain. The SDS has demonstrated reliability and validity in several populations [67–69].

#### Psychological distress

The Profile Of Mood States (POMS) is the ‘gold standard’ assessment for measuring fluctuations in general psychological distress in clinical and research settings [70]. The POMS-Brief [71] was chosen for this study, as it has demonstrated similarly strong internal reliability, validity and specificity to the 65-item original scale [72]. The POMS-Brief asks respondents to rate the extent to which they are experiencing/have experienced 30 affective states on a 5-point Likert scale ranging from 0 *Not at all* to 4 *Extremely* either according to ‘right now’ or ‘past week’ instructions. In the current study, participants responded to the ‘right now’ instruction, in order to assess their affective state directly before, directly after and one week after completing a shift on the crisis line. The measure generates six subscales of five items, representing five domains of negative affect (tension-anxiety, depression-dejection, anger-hostility, fatigue-inertia, confusion- bewilderment), and one domain of positive affect (vigor-activity). A Total Mood Disturbance (TMD) score is calculated by subtracting the score on the vigor-activity subscale from the sum of the other five subscales. Higher scores are indicative of greater mood disturbance. Cronbach’s alpha values for the current sample indicated adequate internal consistency (Time 1 α = .77, Time 2 α = .80, Time 3 α = .75).

The Depression Anxiety Stress Scale– 21 item version [73] is a self-report survey instrument used to measure symptoms of depression, anxiety and stress over the past week. The 21 item version of the scale was selected for the current study on the basis of demonstrated similarly high internal consistency and strong psychometric properties to the longer 42 item version of the DASS in community and clinical populations [73–75], while taking half the time to administer. Participants completed the measure directly before (Time 1) and one week after completing their shift on the crisis line (Time 3). Consistent with scoring instructions, subscale scores of 0–9 indicated *Normal*, 10–13 *Mild*, 14–20 *Moderate*, 21–27 *Severe* and 28 or greater *Extremely severe* symptoms of depression. Subscale scores of 0–7 indicated *Normal*, 8–9 *Mild*, 10–14 *Moderate*, 15–19 *Severe*, and 20 or greater *Extremely severe* symptoms of anxiety. Subscale scores of 0–14 indicated *Normal*, 15–18 *Mild*, 19–25 *Moderate*, 26–33 *Severe* and 34 or greater *Extremely severe* symptoms of stress. In the current study, Cronbach’s alpha values for depression (Time 1 α = .83; Time 3 α = .81, anxiety (Time 1 α = .65; Time 3 α = .73) and stress subscales (Time 1 α = .82; Time 3 α = .78) indicated acceptable internal consistency.

Suicidal ideation was measured by the eight critical items of the Adult Suicidal Ideation Questionnaire [76]: 1) ‘I thought about killing myself’, 2) ‘I thought about how I would kill myself’, 3) ‘I thought about when I would kill myself’, 4) ‘I thought about what to write in a suicide note’, 5) ‘I thought about writing a will’, 6) ‘I thought about telling people I plan to kill myself’, 7) ‘I thought how easy it would be to end it all’, 8) ‘I thought if I had the chance I would kill myself’. These items examine the intensity and lethality of current suicidal thoughts, together with the specificity and availability of a suicide plan in the last month [76]. Responses were made on a 7-point Likert scale ranging from 0 *I have never had this thought before* to 6 *Almost every day*. Individual items scores were summed to obtain a scale score ranging from 0–48, with higher scores indicating more severe suicidal ideation. Scale scores of 0 to 8 indicated *Minimal*, 9–32 *Moderate*, and 33–48 *Critical* suicidal ideation [76, 77]. A Cronbach alpha coefficient of .89 was obtained for the eight items in the current sample, showing good internal consistency.

Two subscales of the Professional Quality of Life scale–Version 5 [78] were used to measure burnout and secondary traumatic stress. The ProQOL conceptualises compassion fatigue as being comprised of two subscales of 10 items each. Burnout (BO) is regarded as feelings of hopelessness and difficulty dealing with work or doing a job effectively (e.g., ‘I feel trapped by my job as a helper’). Secondary traumatic stress (STS) is regarded as a fright response resulting from exposure to traumatic events (e.g., ‘I think that I might have been affected by the traumatic stress of those I help’). Using a 5-point Likert scale ranging from 1 *Never* to 5 *Very often*, respondents are asked to indicate how frequently each item was experienced in the previous week. Higher subscale scores indicate greater risk of BO and STS, respectively. Current scoring guidelines are based on a conservative quartile method with cut-off scores set at the 25th and 75th percentiles to indicate relative risk or protective factors [78]. Convergent and discriminant validity have been established for the scales, and good construct validity is based on more than 200 peer-reviewed articles. Internal consistency coefficients for BO were α = .58 (Time 1) and α = .61 (Time 3), and STS were α = .66 (Time 1) and α = .77 (Time 3).

#### Personal factors

The survey included continuous items to assess participants’ age and number of years of experience as a TCS. Binary items were used to assess participants’ sex (0 = male, 1 = female), relationship status (0 = single/divorced/separated/widowed, 1 = in a relationship/married/de facto), highest educational qualification (0 = no university degree, 1 = university degree), country of birth (0 = Australia, 1 = other), centre location (0 = metropolitan, 1 = inner regional), accreditation level (0 = probationary, 1 = accredited), nature of role/s at Lifeline (0 = volunteer, 1 = paid), number of role/s at Lifeline (0 = 1, 1 = more than 1 role), usual shift frequency (0 = once per week or less, 1 = once per fortnight or more), and time elapsed since most recent shift on the crisis line (0 = 1 week ago or less, 1 = 2 weeks ago or more).

Lived experience of mental health issues was measured using an item used in a previous study [28]. In response to the prompt “Have you ever had a diagnosed mental illness?” participants are asked to select “Yes–within the past 12 months”, “Yes–but not within the past 12 months” or “No”. Those who selected either “Yes” option were asked to specify their diagnosis/es.

Lived experience of suicide was measured by four items of the Suicidal Behaviours Questionnaire–Revised [79] which assess: 1) lifetime suicide ideation and/or suicide attempt, 2) 12 month frequency of suicidal ideation, 3) threat of suicide attempt, and 4) self-reported likelihood of future suicidal behaviour. Items scores were summed to create a score ranging from 3 to 18. In keeping with Osman and colleagues’ scoring instructions, a cut-off score of 7 was used to determine clinically significant suicide risk [79]. The SBQ-R has demonstrated acceptable internal reliability and criterion validity within a range of adult clinical and non-clinical samples [79]. The item “Have you ever been bereaved or affected by another person’s suicide?” was used to assess bereavement by suicide, to which participants answered ‘Yes’ or ‘No’.

Motivations for volunteering as a telephone crisis support workers were measured by the 30-item Volunteer Functions Inventory [80]. Six subscales of five items measure motivations to volunteer as a means of preparing for a new career or maintaining career-relevant skills (career), to obtain satisfactions related to personal growth and self-esteem (enhancement), to reduce guilt over being more fortunate than others and to address one’s own personal problems (protective), an opportunity to be with one’s friends or engage in an activity viewed favourably by others (social), to experience new learning and the chance to use otherwise unpractised knowledge, skills and abilities (understanding), or an opportunity to express values related to altruistic and humanitarian concerns for others (values). Respondents are asked to rate how important each item is as a reason for volunteering as a TCS on a 7-point scale ranging from 1 *Not at all important/accurate* to 7 *Extremely important/accurate*. A series of six studies demonstrated that the VFI yields a theoretically-consistent factor structure, with acceptable internal consistency, temporal stability and predictive validity [80]. Cronbach’s alpha values indicated adequate internal reliability within each subscale: Career α = .93, enhancement α = .85, protective α = .77, social α = .76, understanding α = .74, values α = .73.

#### Shift factors

Consistent with existing service practices, participants answered a number of categorical items in response to each call taken during their shift on the crisis line. They recorded the length of each call, whether the call was a crisis or non-crisis call, the issue which was the focus of the call, whether the caller’s suicidality was discussed, whether a safeplan was created with the caller, whether the caller was at imminent risk of suicide, whether any safety issues (e.g., domestic and family violence, non-suicidal self-injury, child protection) were discussed, and whether the caller was at imminent risk of these safety issues. The Time 2 online survey also included items to assess the number of TCSs and other staff members present during the shift, and whether help was sought from an ISS during the shift.

#### Coping strategies

The Brief COPE [81] was used to measure 14 different coping strategies used in response to a shift on the crisis line: self-distraction (e.g., ‘turning to work or other activities to take my mind off things’), active coping (e.g., ‘taking action to try to make the situation better’), denial (e.g., ‘refusing to believe that it has happened’), substance use (e.g., ‘using alcohol or other drugs to help me get through it’), use of emotional support (e.g., ‘getting comfort and understanding from someone’), use of instrumental support (e.g., ‘getting help and advice from other people’), behavioural disengagement (e.g., ‘giving up trying to deal with it’), venting (e.g., ‘saying things to let my unpleasant feelings escape’), positive reframing (e.g., ‘looking for something good in what is happening’), planning (e.g.,. ‘trying to come up with a strategy about what to do’), humour (e.g., ‘making fun of the situation’), acceptance (e.g., ‘learning to live with it’), religion (e.g., ‘trying to find comfort in my religion or spiritual beliefs’), and self-blame (e.g., ‘criticizing myself’). Participants were asked to rate how frequently they used each strategy to cope with thoughts and feelings about the shift on a 4-point Likert scale ranging from 1 *I haven’t been doing this at all* to 4 *I’ve been doing this a lot*. Cronbach’s alpha values indicated adequate internal reliability within each two-item subscale: Self-distraction α = .68, active coping α = .77, denial α = .54, substance use α = .94, use of emotional support α = .70, use of instrumental support α = .78, behavioural disengagement α = .66, venting α = .51, positive reframing α = .68, planning α = .81, humour α = .66, acceptance α = .56, religion α = .66, self-blame α = .55.

#### Help-seeking

A modified version of the Actual Help-Seeking Questionnaire [82] was used to assess participants’ help-seeking behaviours during the week following their shift on the crisis line. Participants responded *Yes* or *No* to the prompt ‘Have you sought help from any of the following in the past week because of thoughts and feelings about your shift on the crisis line one week ago?’ for the following formal and informal help-sources: intimate partner, friend, parent, other relative/family member, fellow Lifeline TCS, Lifeline In Shift Support person (ISS), colleague from other place of work, Lifeline’s telephone crisis line, other telephone helpline or online chat service, doctor/GP, mental health professional, minister/religious leader, mental health website. The option ‘I did not seek help from anyone’ was also given.

### Data analysis

All statistical analyses were performed with the IBM Statistical Package for the Social Sciences (IBM-SPSS; Chicago, IL, USA) for Windows version 21. Means, standard deviations and bivariate correlations were calculated for study variables. Repeated measures analyses of variance (ANOVA) were used to identify changes in psychological distress and impairment across time points. Structural equation modelling in AMOS was used to test relationships within the hypothesised models of impairment, building up to testing the fully hypothesised models (Fig 1). Moderation relationships were tested using PROCESS, a computational tool for path analysis-based moderation in the form of a conditional process model that uses an ordinary least squares or logistic based path analytical framework to test for both direct and indirect effects [83]. Analyses were conducted according to the specifications set out by PROCESS for SPSS using model one [83], with interaction coefficients reported.

## Results

### Preliminary results

#### Personal factors

Almost one third of participants (*n* = 35, 31.8%) had been diagnosed with a mental illness at some point. Three participants (2.7%) had received a diagnosis within the past 12 months. Diagnoses included anxiety, depressive, bipolar, trauma/stressor-related, eating, personality and substance-related disorders.

Twenty-six participants (23.6%) reported previous suicidal ideation and/or attempt, or self-reported likelihood of future suicidal behaviour indicative of clinically significant suicide risk. Forty-two participants (38.2%) had thought of suicide, and twenty-seven participants (24.5%) had a plan to kill themselves at least once. Twenty-four participants (21.8%) had, at some point, told someone that they were going to suicide. Nineteen participants (17.2%) thought about killing themselves within the past year. Thirteen (11.8%) of these participants had thought about killing themselves rarely (1 time), 4(3.6%) ‘sometimes’ (2 times), 1(0.9%) ‘often’ (3–4 times), and 1 participant (0.9%) had thought about killing themselves ‘very often’ (5 or more times) within the past year. Thirty-nine participants (35.5%) reported that there was a chance they would suicide someday. Thirty-six of these participants (32.8%) reported that they were ‘unlikely’, while 3 participants (2.7%) reported that they were ‘likely’ to suicide someday. The majority of participants (*n* = 61, 55.5%) had been bereaved by suicide at some point.

As a group, participants were most likely to endorse motivations related to values (*M* = 29.92, *SD* = 4.53) and understanding (*M* = 26.18, *SD* = 5.19), followed by enhancement (*M* = 16.46, *SD* = 6.92), career (*M* = 14.75, *SD* = 8.76), protective (*M* = 12.05, *SD* = 5.63) and social motivations for volunteering as a TCS (*M* = 11.76, *SD* = 5.70).

#### Shift factors

On average, participants reported that there were two other TCSs (*M* = 2.39, *SD* = 1.00, Range = 1–5), and two additional people (e.g., In Shift Support worker [ISS], other staff members) (*M* = 2.68, *SD* = 1.54, Range = 1–11) present at the centre during their shift on the crisis line. Approximately half of all participants spoke to their ISS before starting (*n* = 66, 60.0%) and after finishing their shift on the crisis line (*n* = 51, 46.4%). Approximately one quarter of participants spoke to their ISS during (*n* = 33, 30.0%) or after at least one call during their shift (*n* = 30, 27.3%). A significant proportion of participants (*n* = 17, 15.5%) did not speak to their ISS before, during or after their shift on the crisis line.

On average, participants took seven calls during their shift (*M* = 6.82, *SD* = 2.46). Participants spent an average of 115 minutes speaking with callers (*M* = 114.79, *SD* = 32.59, Range = 53–182) during their shift. Calls primarily focused on family and relationship issues (17.1%), issues related to the self (15.3%), mental health (12.3%), general health (5.5%) and suicide (3.8%). Suicide was specifically discussed in just over half of all calls (56.5%), and was imminent in 1% of calls. A significant proportion of calls (16.8%) involved safety issues (e.g., domestic violence, child protection, third party suicide, crime/other emergency), which were imminent in 3.2% of calls. The majority of participants (56.5%) took at least one call which involved one or more safety issues. A significant proportion of participants (17.7%) spoke to one or more callers for whom at least one safety issue presented an imminent risk.

#### Coping strategies

As a group, participants used acceptance (*M* = 3.07, *SD* = 1.31) and positive reframing strategies (*M* = 3.02, *SD* = 1.26) ‘a medium amount’, and emotional support (*M* = 2.94, *SD* = 1.25), active coping (*M* = 2.69, *SD* = 1.24), religion (*M* = 2.56, *SD* = 1.00), self-distraction (*M* = 2.54, *SD* = 1.06), venting (*M* = 2.50, *SD* = .73), instrumental support (*M* = 2.48, *SD* = .93), planning (*M* = 2.48, *SD* = .87), self-blame (*M* = 2.39, *SD* = .67), humour (*M* = 2.33, *SD* = .71), substance use (*M* = 2.11, *SD* = .63), behavioural disengagement (*M* = 2.03, *SD* = .28) and denial strategies (*M* = 2.02, *SD* = .21) ‘a little bit’ to cope with thoughts and feelings about their shift during the week after completing a shift on the crisis line.

### Help-seeking

Just under one half (*n* = 51, 46.4%) of participants did not seek help from anyone during the week following their shift on the crisis line. Participants who did seek help preferred to speak to an intimate partner (*n* = 24, 21.8%), followed by an ISS (*n* = 16, 14.5%), friend (*n* = 10, 9.1%), fellow TCS (*n* = 7, 6.4%), relative (*n* = , 3.6%), or mental health professional (*n* = 2, 1.8%).

#### Affective response

As a group, participants reported significantly higher total mood disturbance directly after than directly before completing a shift on the crisis line (Table 2). Participants reported significantly greater tension-anxiety and confusion-bewilderment directly before than directly after completing a shift. They reported significantly greater fatigue-inertia directly after than directly before the shift, which did not reduce significantly during the week after completing a shift on the crisis line. Participants reported significantly lower levels of vigor-activity directly after compared to directly before completing a shift on the crisis line. They also reported significantly lower levels of vigor-activity one week after compared to directly before completing a shift. Participants reported similar levels of depression-dejection and anger-hostility across time points.

**Table 2:** Affective responses directly before, directly after and one week after completing a shift on the crisis line.

	Directly before *M(SD)*	Directly after *M(SD)*	One week after *M(SD)*	*F*	*P*
Fatigue-inertia	3.02(3.10)^a^	4.15(4.04)^a^	3.60(3.67)	5.30	.007
Confusion-bewilderment	2.43(1.73)^b^	1.85(1.40)^b^	2.27(1.57)	6.89	.002
Tension-anxiety	2.17(2.60)^c^	1.13(2.16)^c^	1.31(1.83)	12.10	.000
Depression-dejection	1.00(2.11)	.67(1.57)	.99(1.85)	1.83	.167
Anger-hostility	.24(.70)	.34(1.22)	.42(.82)	2.02	.139
Vigor-activity	6.97(3.89)^de^	3.91(3.73)^df^	5.73(4.17)^ef^	29.94	.000
TMD	1.87(9.94)^g^	4.22(9.38)^g^	2.81(9.94)	4.68	.012

*Note*. TMD = Total Mood Disturbance. Means in the same row with the same subscript letter are significantly different.

#### Psychological distress

Most participants reported minimal suicidal ideation (n = 114, 91.9%), while four participants (3.2%) reported moderate suicidal ideation during the month before completing a shift on the crisis line. Most participants reported normal to mild symptoms of depression, anxiety and stress, while a significant proportion reported moderate to extremely severe symptoms of depression, anxiety and stress during the week before and the week after completing a shift on the crisis line (Table 3). All participants reported low symptoms of burnout and secondary traumatic stress at each time point (Table 3). Participants reported significantly more severe symptoms of anxiety, stress and secondary traumatic stress during the week before compared to the week after completing a shift on the crisis line (Table 3). Symptoms of depression and burnout did not differ significantly from the week before to the week after completing a shift (Table 3).

**Table 3:** Symptoms of psychological distress during the week before and the week after completing a shift on the crisis line (*N* = 110).

	Symptoms during the week before completing a shift			Symptoms during the week after completing a shift			*F*	*p*
	*M(SD)*	Normal/Minimal/Mild/Low *n*(%)	Moderate *n*(%)	*M(SD)*	Normal/Minimal/Mild/Low *n*(%)	Moderate *n*(%)		
Depression	3.35(4.86)	90(81.8)	14(12.7)	2.72(3.96)	82(74.5)	10(9.0)	2.97	.088
Anxiety	2.25(3.15)	87(79.1)	17(15.4)	1.42(3.03)	85(77.2)	7(6.3)	8.48	.005
Stress	7.01(5.83)	67(60.9)	37(33.7)	5.87(5.26)	72(65.4)	20(18.1)	5.47	.022
BO	17.09(3.37)	102(92.7)	-	16.99(3.33)	92(83.6)	-	.19	.663
STS	15.81(3.30)	102(92.7)	-	15.09(3.83)	92(83.6)	-	5.89	.017

*Note*. BO = Burnout. STS = Secondary traumatic stress.

Frequencies for each symptom type at each time point do not add to 100% where some participants chose not to complete symptom measures.

### Intercorrelations

Correlations supported hypothesised relationships between model variables. Significant correlations were found between all affective response variables with psychological distress during the week before and the week after completing a shift on the crisis line (H1). Personal factors age, education, accreditation status, shift frequency, lived experience of mental health, lived experience of suicide, bereaved by suicide, career motivations, values motivations, protective motivations, understanding motivations (H2) and shift factors number of TCS present, number of others present, total length of calls taken, no contact with ISS, number of calls taken with a focus on carer issues (H3) correlated significantly with psychological distress variables. Significant correlations were found between all psychological distress variables and at least one impairment variable (H4). Help-seeking from a partner, friend or ISS (H5) and the coping strategies of self-distraction, active coping, behavioural disengagement, venting, planning, religion and self-blame (H6) were significantly correlated with at least one impairment variable. Remaining study variables did not correlate significantly, and as such were not included in the models.

### Main results

#### Level of impairment before and after shift

On average, participants reported that their symptoms of psychological distress caused impaired their functioning on less than one day during the week before and the week after completing a shift on the crisis line (Table 4). However, frequency statistics indicated that a small proportion of participants reported a high level of disruption to their normal TCS, work/study, home/family, and social/leisure activities during the week before and the week after completing a shift on the crisis line. A significant proportion of participants reported that they were totally unable to manage, or had to cut down their normal activities within each of these functional domains during the week before and the week after completing a shift.

**Table 4:** Impairment during the week before and after completing a shift on the crisis line.

Week before shift					Week after shift			
	Disruption *M*	High disruption *n*(%)	At least 1 day missed *n*(%)	At least 1 day productivity reduced *n*(%)	Disruption *M*	High disruption *n*(%)	At least 1 day missed *n*(%)	At least 1 day productivity reduced *n*(%)
Home/family	.95^bc^	8(7.2)	13(11.8)	21(19.1)	.70	5(4.5)	8(7.2)	19(17.3)
Work/ study	.79^a^	4(3.6)	6(5.4)	18(16.3)	.53	2(1.8)	4(3.6)	16(14.5)
Social/ leisure	.60^c^	4(3.6)	16(14.5)	16(14.5)	.55	3(2.7)	7(6.3)	12(10.9)
TCS	.41^1ab^	3(2.7)	4(3.6)	2(1.8)	.72^1^	2(1.8)	5(4.5)	6(5.5)

*Note*. Mean values for disruption in the same column with the same subscript letter are significantly different. Mean values for disruption in the same row with the same subscript number are significantly different. ***p < .001, **p < .01, *p < .05.

Participants reported significantly greater disruption to TCS activities during the week after compared to the week before completing a shift on the crisis line, *F*(1, 88) = 7.51, *p* = .007. Disruption to work/study, home/family and social/leisure activities did not differ significantly from the week before to the week after completing a shift. During the week before completing a shift on the crisis line, participants reported a significantly lower level of disruption to TCS activities compared to work/study activities, and home/family activities. Disruption to home/family activities was significantly greater than disruption to social/leisure activities during the week before completing a shift. Levels of disruption across different functional domains did not differ significantly during the week after completing a shift on the crisis line.

### Disruption to TCS activities

#### Week before shift

**Fig 2:**
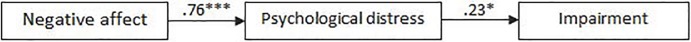
Empirically supported model of disruption to TCS related activities during the week before completing a shift on the crisis line.

The coefficients between TMD and distress (*β* = .76, *p* = .000) and distress and impairment (*β* = .23, *p* = .038) were significant, and model fit was adequate (*χ*^2^ = 38.49, *p* = .008, CFI = .91, RMSEA = .08), supporting Hypotheses 1 and 2 (see Fig 2). No support was found for Hypothesis 3, as the coefficient between personal factors and distress was not significant (*β* **=** -.09, *p* = .344).

#### Week after shift

**Fig 3:**
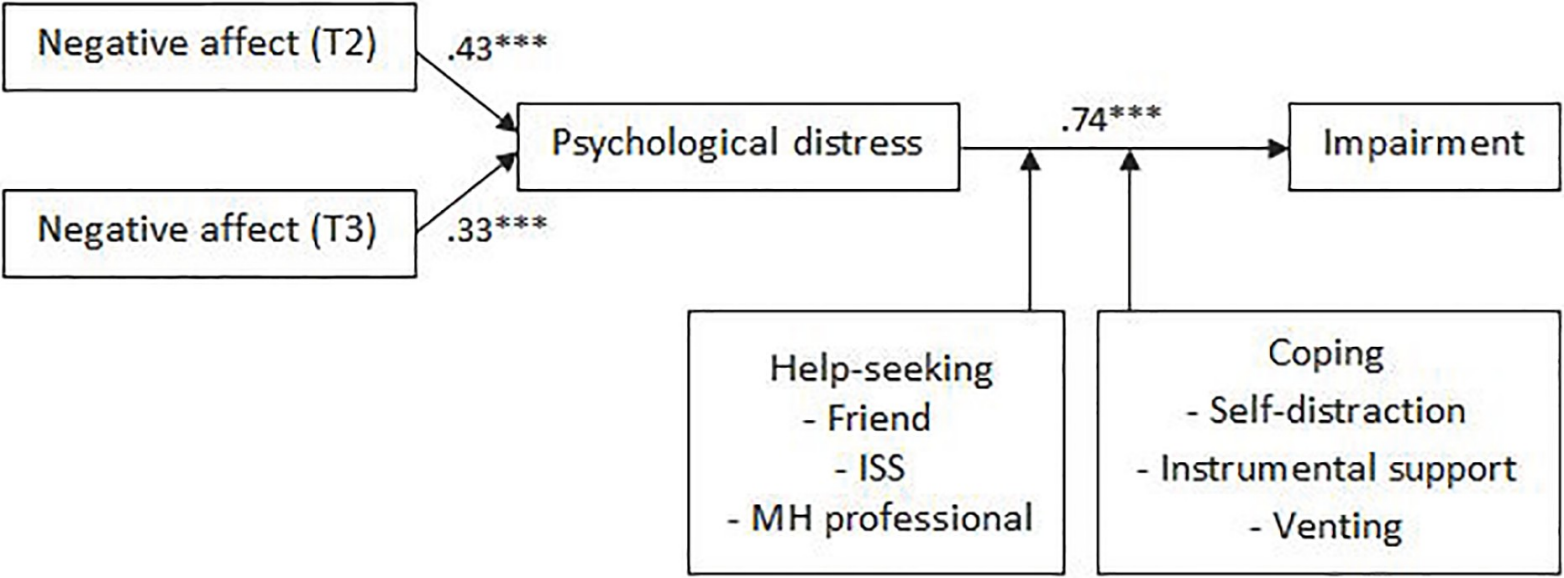
Empirically supported model of disruption to TCS related activities during the week after completing a shift on the crisis line.

The coefficients between TMD and distress (TMDT2 *β* = .43, *p* = .000; TMDT3 *β* = .33, *p* = .000) and distress and impairment (*β* = .74, *p* = .000) were significant, and model fit was adequate (*χ*^2^ = 36.58, *p* = .009, CFI = .94, RMSEA = .08, supporting Hypotheses 1 and 2 (see Fig 3). Coefficients between personal factors and distress (*β* = -.04, *p* = .622) and shift factors and distress (*β* = .25, *p* = .137) were not significant, finding no support for Hypotheses 3 and 4. Partial support was found for Hypotheses 5 and 6, as seeking help from a friend (*B* = .05, *p* = .023), an ISS (*B* = .06, *p* = .005), or mental health professional (*B* = -.20, *p* = .006), using self-distraction (*B* = .01, *p* = .013), instrumental support (*B* = .04, *p* = .001), and venting (*B* = .02, *p* = .007) significantly moderated the relationship between distress and impairment.

### Disruption to work/study activities

#### Week before shift

**Fig 4:**
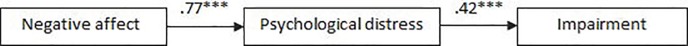
Empirically supported model of disruption to work/study activities during the week before completing a shift on the crisis line.

The coefficients between TMD and distress (*β* = .77, *p* = .000) and distress and impairment (*β* = .42, *p* = .000) were significant, and model fit was adequate (*χ*^2^ = 40.23, *p* = .005, CFI = .91, RMSEA = .08), supporting Hypotheses 1 and 2 (Fig 4). Hypothesis 3 was not supported, as the coefficient between personal factors and distress was not significant (*β* **=** -.14, *p* = .130).

#### Week after shift

**Fig 5:**
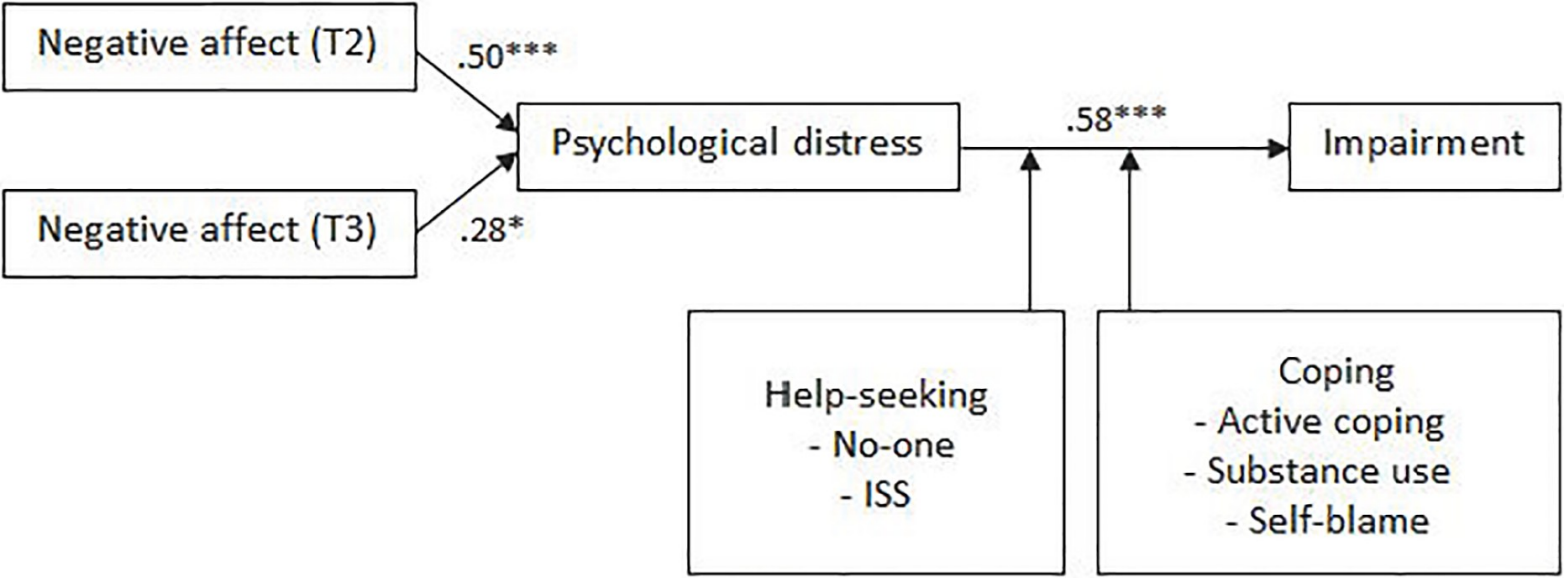
Empirically supported model of disruption to work/study activities during the week after completing a shift on the crisis line.

Coefficients between TMD and distress (TMDT2 *β* = .50, *p* = .000; TMDT3 *β* = .28, *p* = .017) and distress and impairment (*β* = .58, *p* = .000) were significant, supporting Hypotheses 1 and 2 (Fig 5). Model fit was adequate (*χ*^2^ = 40.10, *p* = .003, CFI = .92, RMSEA = .08). No support was found for Hypotheses 3 or 4, as the coefficients between personal factors and distress (*β* **=** -.05, *p* = .565) and shift factors and distress (*β* = .38, *p* = .061) were not significant. Hypothesis 5 was partially supported, as not seeking help from anyone (*B* = -.07, *p* = .002), and seeking help from an ISS (*B* = .06, *p* = .011) significantly moderated the relationship between distress and impairment. Partial support was found for Hypothesis 6, as the relationship between distress and impairment was significantly moderated by active coping (*B* = -.02, *p* = .043), substance use (*B* = -.03, *p* = .024), religion (*B* = .03, *p* = .001) and self-blame coping strategies (*B* = -.03, *p* = .010).

### Disruption to home/family activities

#### Week before shift

**Fig 6:**
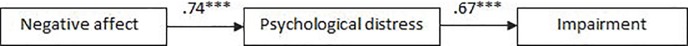
Empirically supported model of disruption to home/family activities during the week before completing a shift on the crisis line.

Coefficients between TMD and distress (*β* = .74, *p* = .000) and distress and impairment (*β* = .67, *p* = .000) were significant, supporting Hypotheses 1 and 2 (Fig 6). Model fit was not adequate (*χ*^2^ = 60.93, *p* = .000, CFI = .84, RMSEA = .14). No support was found for Hypothesis 3, as the coefficient between personal factors and distress was not significant (*β* **=** -.10, *p* = .268).

#### Week after shift

**Fig 7:**
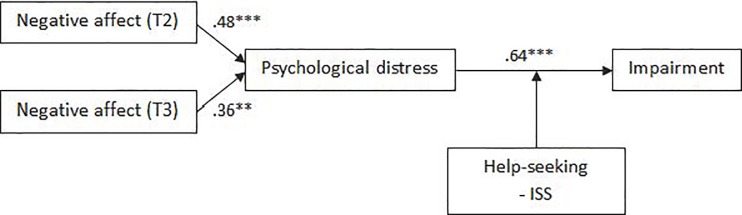
Empirically supported model of disruption to home/family activities during the week after completing a shift on the crisis line.

Coefficients between TMD and distress (T2 *β* = .48, *p* = .000; T3 *β* = .36, *p* = .002) and distress and impairment (*β* = .64, *p* = .000) were significant, and model fit was adequate (*χ*^2^ = 31.87, *p* = .032, CFI = .95, RMSEA = .07), supporting Hypotheses 1 and 2 (Fig 7). No support was found for Hypotheses 3 or 4, as the coefficients between personal factors and distress (*β* **=** -.04, *p* = .627) and shift factors and distress (*β* = .36, *p* = .072) were not significant. Partial support was found for Hypotheses 5, as the relationship between distress and impairment was significantly moderated by seeking help from an ISS (*B* = .07, *p* = .010). Hypothesis 6 was not supported, as coping strategies did not moderate the relationship between distress and impairment.

### Disruption to social/leisure activities

#### Week before shift

**Fig 8:**
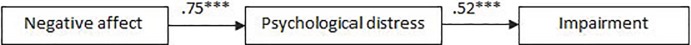
Empirically supported model of disruption to social/leisure activities during the week before completing a shift on the crisis line.

Coefficients between TMD and distress (*β* = .75, *p* = .000) and distress and impairment (*β* = .52, *p* = .000) were significant, supporting Hypotheses 1 and 2 (Fig 8). Model fit was adequate (*χ*^2^ = 49.70, *p* = .001, CFI = .90, RMSEA = .08). No support was found for Hypothesis 3, as the coefficient between personal factors and distress was not significant (*β* **=** -.09, *p* = .330).

#### Week after shift

**Fig 9:**
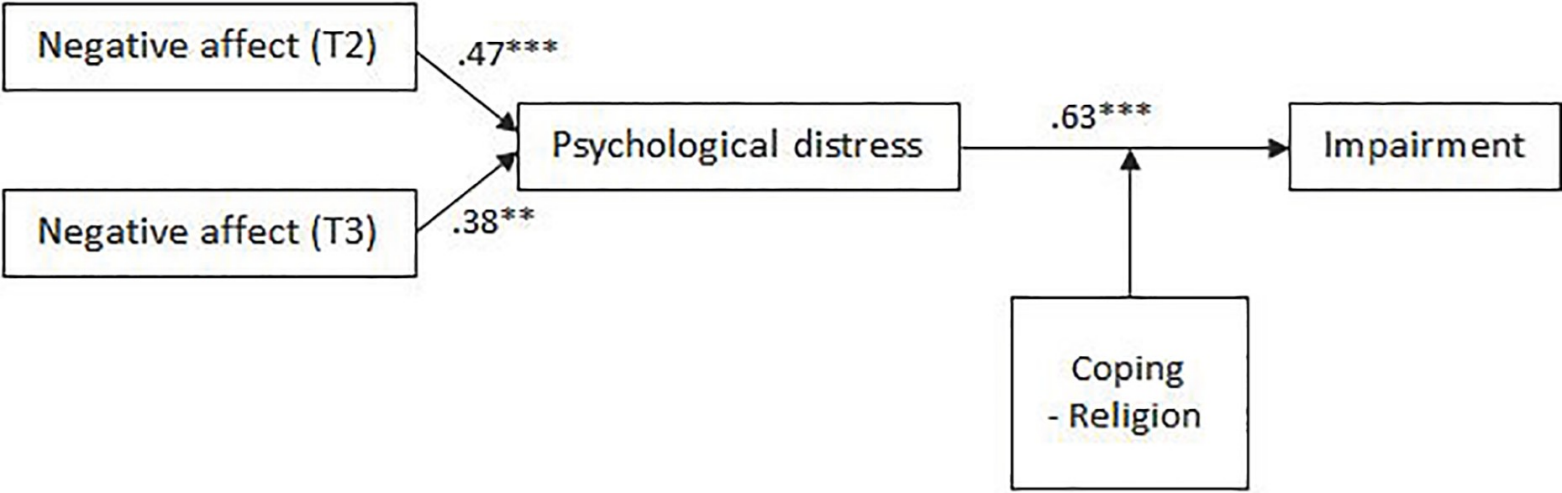
Empirically supported model of disruption to social/leisure activities during the week after completing a shift on the crisis line.

Coefficients between TMD and distress (T2 *β* = .47, *p* = .000; T3 *β* = .38, *p* = .001) and distress and impairment (*β* = .63, *p* = .000) were significant, and model fit was adequate (*χ*^2^ = 32.82, *p* = .025, CFI = .95, RMSEA = .08), supporting Hypotheses 1 and 2 (Fig 9). No support was found for Hypothesis 3 or 4, as the coefficients between personal factors and distress (*β* **=** -.05, *p* = .533) and shift factors and distress (*β* = .36, *p* = .067) were not significant. Hypothesis 5 was not supported, as help-seeking did not moderate the relationship between distress and impairment. Partial support was found for Hypothesis 6, as the relationship between distress and impairment was significantly moderated by seeking using religion to cope (*B* = .03, *p* = .020).

## Discussion

Telephone crisis lines are effective in attracting those with mental health issues and those experiencing suicidality; they continue to be accessed by help-seekers at an increasing rate. However, little is known about how the workforce involved in the delivery of telephone crisis lines is performing, including whether or not they are resilient to the inevitable occupational hazards of this service environment. The current study aimed to measure telephone crisis support workers’ functional impairment during the week before and the week after completing a shift on the crisis line, and to identify mechanisms of impairment.

A significant number of telephone crisis support workers who participated in this study reported impaired functioning during the week before and the week after completing a shift on the crisis line. As expected, negative affect predicted functional impairment via symptoms of psychological distress. This was the case for impairment within each functional domain during the week before and the week after completing a shift on the crisis line. Also as expected, seeking help for and using particular strategies to cope with thoughts and feelings about the shift moderated the relationship between psychological distress and impairment. These results are consistent with the literature on helping professionals and telephone crisis support workers, which suggests that strong negative affective responses to others in distress are likely to lead to psychological distress and functional impairment in the absence of help-seeking or the use of adaptive coping strategies [19, 28, 53]. Seeking help from an In Shift Support worker, or using self-distraction, instrumental support, venting, substance use, religion or self-blame as coping strategies significantly increased the effect of psychological distress on impairment, suggesting that using each of these strategies alone is not sufficient to prevent functional impairment from occurring. Conversely, seeking help from a mental health professional and using active coping strategies (e.g., problem solving) significantly reduced the effect of psychological distress on impairment, highlighting these as adaptive strategies to prevent the progression from symptoms of psychological distress to functional impairment.

During the week before completing a shift, participants reported significantly greater disruption to TCS activities than to work/study or family/home activities, and significantly greater disruption to family/home activities than to social/leisure activities. They also reported significantly greater disruption to TCS activities during the week after compared to the week before completing a shift on the crisis line. These results may indicate that TCSs prioritise their work/study and particularly their family/home activities over TCS-related activities during the week before completing a shift, and are less able to participate in TCS activities during the week after compared to the week before completing a shift on the crisis line. Future research is needed to confirm and explain these possibilities.

Contrary to expectations, most participants reported symptoms of psychological distress which fell within the normal range. It is possible that this is a true reflection of participants’ symptoms, or that participants were not able to identify and self-report their symptoms. However, in the context of the literature on helping professionals’ reluctance to self-report personal symptoms of psychological distress [15, 59, 60], it seems more likely that participating telephone crisis support workers under-reported their symptoms in order to be perceived as competent to continue supporting callers. Nevertheless, a significant number of participants did report moderate suicidal ideation, and moderate to extremely severe symptoms of depression, anxiety and stress, and these participants experienced significantly greater impairment across all functional domains during the week before and the week after completing a shift than those who reported low level symptoms.

Participants reported significantly greater negative affect directly after than directly before completing a shift. Specifically, they reported significantly greater fatigue-inertia directly after than directly before the shift, which did not reduce significantly during the week. These results lend support to neuroscientific research suggesting that those who engage empathically with others in distress will experience similar negative affective reactions which increase their risk for personal symptoms of psychological distress [32, 35–37]. As a group, participants reported significantly greater vigor-activity and tension-anxiety directly before than directly after or one week after completing a shift on the crisis line. Taken together with the fact that participants reported significantly more severe symptoms of anxiety, stress and secondary traumatic stress during the week before compared to the week after completing a shift on the crisis line, this suggests that psychological distress is also experienced in anticipation of the shift, which may relate to revisiting previous calls as preparation for shift performance. However, further research is needed to examine this possibility.

Contrary to expectations, the inclusion of personal variables and shift factors did not significantly improve model fit. It is possible that there are other, more important factors associated with telephone crisis support workers’ symptoms of psychological distress that were not captured in this study. However, the model including negative affective responses to empathic engagement with crisis callers, symptoms of psychological distress, help-seeking and coping did provide an adequate fit to the data, suggesting that these are the primary mechanisms of telephone crisis support workers’ functional impairment. This is an important finding because, while personal variables and shift factors are not readily modifiable, it is possible to reduce the impact of affective responses to empathic engagement, and to facilitate appropriate help-seeking and the use of adaptive coping strategies in order to prevent functional impairment occurring in response to symptoms of psychological distress.

A large number of participating telephone crisis support workers reported lived experience of suicide. One quarter of participants reported previous suicidal ideation and/or attempt, or self-reported likelihood of future suicidal behaviour indicative of clinically significant suicide risk. More than one third of participants had thought of suicide at some point. One quarter of participants had a plan to kill themselves at least once. More than one quarter of participants had thought about killing themselves within the past year. More than half of all participants identified as being bereaved by suicide. Participants who experienced clinically significant suicide risk reported more severe symptoms of suicidal ideation, symptoms of stress, depression, burnout and secondary traumatic stress than those who did not. Participants who identified as suicide bereaved reported significantly more severe symptoms of burnout than those who did not.

A number of limitations of this study are worth noting. Firstly, data were provided by a representative sample of telephone crisis support workers from one organisation. Further studies with workers from other organisations are needed to determine population validity. A second potential limitation of the study is the role of self-reporting biases such as social desirability. While they were not suited to the current large-scale investigation required to detect direct and indirect relationships between functional impairment and other variables collectively, future studies may utilise observation and clinician-rated scales rather than self-report measures. Thirdly, questions about causality have not been adequately answered by the analyses presented in this study. Longitudinal studies are required to uncover causal relationships between model variables.

Despite the aforementioned limitations, this study makes a number of unique contributions to the literature. By employing a repeated-measures design and domain-specific measure of functional impairment, this study has provided specific information regarding telephone crisis support workers’ impairment within different functional domains before and after completing a shift on the crisis line. By extending a previous model of telephone crisis support workers’ functional impairment to include additional mechanisms, direct and indirect relationships between a larger networks of variables than in previous studies were able to be examined simultaneously. An important outcome of this study is empirically-supported models of telephone crisis support workers’ functional impairment within their role as a TCS, as well as in relation to their other work/study, home/family and social/leisure activities. However, further research is required to delineate in greater detail the process by which telephone crisis support workers become impaired. Qualitative research may be particularly well-suited to this endeavour, and to informing the modification or development of service strategies to optimise telephone crisis support workers’ psychological wellbeing and functioning, including their delivery of support to callers.

## Supporting information

S1 Table STROBE checklist. (DOC)
